# Untargeted Lipidomic Approach for Studying Different Nervous System Tissues of the Murine Model of Krabbe Disease

**DOI:** 10.3390/biom13101562

**Published:** 2023-10-23

**Authors:** Husam B. R. Alabed, Ambra Del Grosso, Valeria Bellani, Lorena Urbanelli, Sara Carpi, Miriam De Sarlo, Lorenzo Bertocci, Laura Colagiorgio, Sandra Buratta, Luca Scaccini, Dorotea Frongia Mancini, Ilaria Tonazzini, Marco Cecchini, Carla Emiliani, Roberto Maria Pellegrino

**Affiliations:** 1 Department of Chemistry, Biology and Biotechnology, University of Perugia, 06100 Perugia, Italy; husambr.alabed@studenti.unipg.it (H.B.R.A.); valeria.bellani@studenti.unipg.it (V.B.); lorena.urbanelli@unipg.it (L.U.); lorenzo.bertocci@studenti.unipg.it (L.B.); sandra.buratta@unipg.it (S.B.); dorotea.frongiamancini@studenti.unipg.it (D.F.M.); carla.emiliani@unipg.it (C.E.); 2 NEST (National Enterprise for nanoScience and nanoTechnology), Istituto Nanoscienze-CNR and Scuola Normale Superiore, Piazza San Silvestro 12, 56127 Pisa, Italy; ambra.delgrosso@sns.it (A.D.G.); sara.carpi@nano.cnr.it (S.C.); miriam.desarlo@nano.cnr.it (M.D.S.); lauracolagiorgio@gmail.com (L.C.); luca.scaccini@sns.it (L.S.); ilaria.tonazzini@nano.cnr.it (I.T.); marco.cecchini@nano.cnr.it (M.C.); 3 Centro di Eccellenza sui Materiali Innovativi Nanostrutturati (CEMIN), University of Perugia, Via del Giochetto, 06123 Perugia, Italy; 4 Department of Health Sciences, University “Magna Graecia” of Catanzaro, Viale Europa, Località Germaneto, 88100 Catanzaro, Italy

**Keywords:** lipidomics, LC/MS, Krabbe disease, psychosine, Twitcher mouse, untargeted mass spectrometry

## Abstract

Krabbe disease is a rare neurodegenerative disease with an autosomal recessive character caused by a mutation in the *GALC* gene. The mutation leads to an accumulation of psychosine and a subsequent degeneration of oligodendrocytes and Schwann cells. Psychosine is the main biomarker of the disease. The Twitcher mouse is the most commonly used animal model to study Krabbe disease. Although there are many references to this model in the literature, the lipidomic study of nervous system tissues in the Twitcher model has received little attention. This study focuses on the comparison of the lipid profiles of four nervous system tissues (brain, cerebellum, spinal cord, and sciatic nerve) in the Twitcher mouse compared to the wild-type mouse. Altogether, approximately 230 molecular species belonging to 19 lipid classes were annotated and quantified. A comparison at the levels of class, molecular species, and lipid building blocks showed significant differences between the two groups, particularly in the sciatic nerve. The in-depth study of the lipid phenotype made it possible to hypothesize the genes and enzymes involved in the changes. The integration of metabolic data with genetic data may be useful from a systems biology perspective to gain a better understanding of the molecular basis of the disease.

## 1. Introduction

Globoid cell leukodystrophy, also called Krabbe disease (KD; OMIM #245200), is an autosomal recessive sphingolipidosis belonging to the larger class of lysosomal storage disorders (LSDs). It is characterized by decreased or abolished GALC enzymatic activity within cell lysosomes due to homozygous or compound heterozygous mutations in the galactosyl ceramidase gene (*GALC*, OMIM #606890; chromosome 14q31). Different subtypes of KD, including infantile, late infantile, juvenile, and adult, are recognized based on specific mutations [1]. GALC enzyme deficiency leads to an accumulation of the cytotoxic galactosyl sphingosine (psychosine, PSY) in the myelin sheath. This causes degeneration of oligodendrocytes and Schwann cells and the formation of macrophage-derived, psychosine-rich globoid cells [2]. Enzyme replacement in vivo seems to be insufficient to completely restore homeostasis and can only partially eliminate PSY [3]. Furthermore, a number of findings are appearing in the literature involving new aspects of KD’s progression that cannot be explained by PSY accumulation alone. One of the latest aspects highlighted is that PSY is released in extracellular vesicles in the brain of the Twitcher (TWI) mouse (the natural KD murine model). Changes were found in the structure and function of the brain endothelium of KD patients and TWI mice, in vivo and in vitro, demonstrating an increased permeability of the vessels [4]. In addition, KD seems to be characterized by autophagy dysregulation, neuronal degeneration, and calcium signaling defects. The only available clinical treatment is hematopoietic stem cell transplantation (HSCT) for presymptomatic patients. Experimental treatments are currently under pre-clinical study, such as gene therapy (GT), chaperone-mediated therapy, or nanovector-mediated enzyme replacement therapy (ERT) [1].

Lipids are an important class of biomolecules characterized by hundreds or thousands of molecules with a considerable structural diversity that is reflected in the variety and complexity of the physiological processes in which they are involved (signaling membrane, reserve energy, endocrine action) [5]. Since 2005, the International Lipid Classification and Nomenclature Committee under the sponsorship of the Lipid Metabolites and Pathways Strategy (LIPID MAPS) consortium established the “Comprehensive Classification System for Lipids” that divided all lipids into eight lipid categories: fatty acyls, glycerolipids, glycerophospholipids, sphingolipids, sterol lipids, prenol lipids, saccharolipids, and polyketides. The complete set of lipid species in a cell, tissue, organism, or biological system is referred to by researchers as the “lipidome” and the broad-scale analytic study of pathways and networks of the lipidome as “lipidomics” [6]. Lipidomics is a relatively young field of science that employs a variety of techniques to quantify the hundreds of chemically distinct lipids in cells and to spot changes in lipid metabolism and lipid-mediated signaling pathways that control cellular homeostasis in both health and sickness [7]. Thanks to technological advances over the last decade, which have made it possible to carry out sensitive and accurate lipidomic research, there has been a recent increase in interest in the study of lipid molecules, as is well shown by the increase in the number of research articles published over the past few years [6], and lipidomics is now seen as one of the most important and fundamental “omics”. The characterization of lipid profiles is increasingly conducted through LC/MS (liquid chromatography/mass spectrometry) analyses. Bioinformatics tools like LipidOne [8], Biopan [9], the MetaboAnalist 5.0 web platform [10], and OmicsNet [11] prove to be indispensable for processing data matrices, enabling comparisons between samples and variables. These tools facilitate the study of variations in molecular species and building blocks, as well as the prediction of the biochemical pathways involved [12].

As metabolic diseases can develop long before they become clinically visible, it is particularly important to identify potential biomarkers in order to find suitable treatments or preventive measures for the right patients. Nowadays, lipidomics is becoming a key tool for biomarker research, as thanks to the lipid profiling of potential biomarkers for disease, diagnosis and prognosis can be identified [13]. Indeed, recent research has revealed that any disruption in a biological system is likely to affect its lipid pool’s abundance and/or composition. Furthermore, it has been shown that alterations in the blood lipid profile may be connected to the development of several serious human disorders, including cardiovascular disease [14], hypertensive diseases [15], Alzheimer’s disease [16], and cancer [17]. Despite this, the lipid composition of human or animal models of KD in central and in peripheral nervous system tissues or cells has received no attention in the literature. To the best of our knowledge, no works have been carried out using a lipidomics approach, although it is known that the molecular trigger and biomarker of KD is PSY, a lipid belonging to the class of lysosphingolipid, and lipid metabolism is critical in diseases belong to the class of LSDs, like KD. Only a few works concern the study of PSY, without a lipidomics approach but with biochemical approaches [18,19,20,21,22]. Therefore, the aim of this work is to investigate further lipidomic analyses of nervous system tissues, including sciatic nerve tissue (SN), spinal cord tissue (SC), and brain (B) and cerebellum (C) tissue in both a mouse model of KD (HOM) and healthy wild-type mice (WT), especially to highlight differences between the lipid profiles of healthy and sick subjects. In fact, in the context of systems biology, any alteration in the lipid profile may be linked to alterations in the proteome and modifications in the genome [12].

To carry out our research, we performed an untargeted lipidomic analysis on nervous system tissues of a mouse model of KD using LC/MS Q-TOF and then we proceeded with an analysis of the complex dataset obtained. Any differences will also relate to PSY levels. For this purpose, in addition to the lipid profile, the work proposes the quantification of PSY by means of a targeted approach based on LC/MS Q-TOF.

## 2. Materials and Methods

### 2.1. Samples

Twitcher heterozygous (HET) mice (TWI+/− C57BL6 mice; the Jackson Laboratory, Bar Harbor, ME, USA), kindly provided by Prof. Gritti (San Raffaele Telethon Institute for Gene Therapy, Milan, Italy), were used as breeder pairs to generate homozygous TWI mice (HOM) at the Center for Experimental Biomedicine of CNR, in Pisa. Animals were maintained under standard housing conditions and used according to the protocols and ethical guidelines approved by the Ministry of Health (permit no. 535/2018-PR; official starting date: 9 July 2018). Mice genomic DNA was extracted from clipped tails of post-natal day (PND) 10–15 mice (EUROGOLD Tissue-DNA Mini Kit, EUROCLONE) and the genetic status of each mouse was determined from the genome analysis of the TWI mutation, with a fast real-time PCR-based protocol that we set up in the work of Carpi et al. [23]. HOM and wild-type (WT) animals were used for experiments, while HET littermates were retained for colony maintenance. When HOM mice reached a ponderal weight loss ≥ 25% PND 39–42, which is a very terminal phase of the disease characterized by profound neurodegeneration and demyelination, they were deeply anesthetized with a urethane solution (0.8 mL/Hg; Sigma-Aldrich, St. Louis, MO, USA) and euthanized via transcardial perfusion with PBS. Twitcher mice, in fact, typically die around PND 40–45, as already reported in many research papers by us and others [23,24]. WT mice, instead, were sacrificed with the last sacrificed HOM mouse of the experimental group. Subsequently, the tissues were extracted from each mouse and processed. All procedures were performed with maximal efforts to minimize suffering in the mice.

### 2.2. Preparation and Storage

Tissues (brain, cerebellum, sciatic nerves, and spinal cord) were extracted from each mouse and immediately put into 1.5 mL microcentrifuge tubes on ice. Tubes were previously put on ice with distinct volumes of RIPA buffer (R0278; Sigma Aldrich), depending on the organ (1000 μL for the brain; 300 μL for the cerebellum; 200 μL for the spinal cord; and 100 μL for the sciatic nerves). RIPA buffer was previously prepared with a cocktail of both protease and phosphatase inhibitors (cOmplete (4693116001) and PhosSTOP (4906845001); Roche Diagnostics, Indianapolis, IN, USA). Organs were then homogenized on ice with pestles, sonicated for 5 s, and centrifuged (15,000× *g* for 30 min, at 4 °C). Subsequently, they were tested for protein concentration using the micro-bicinchoninic acid (BCA) Protein Assay Kit (Thermo Scientific Pierce, Waltham, MA, USA). The BCA assay was carried out using 1 μL (for cerebellum, sciatic nerves, and spinal cord) or 0.5 μL (for the brain) of the organ lysate diluted in 100 μL of sterile distilled water, in 96-well plates. Each sample was assayed in duplicate. Absorbance (562 nm) was read using the GloMax^®^ Discover Microplate fluorescence reader (Promega, Madison, WI, USA). Lysates of tissues were stored at −80 °C.

### 2.3. Chemicals and Reagents

Water (LiChrosolv) LC/MS grade, methanol, acetonitrile, and isopropanol were all obtained from Merck KGaA (Darmstadt, Germany), and methyl-tert-butyl ether (MTBE), chloroform (CHCl_3_), ammonium acetate, and ammonium fluoride were all from Sigma-Aldrich (Sigma-Aldrich GmbH, Hamburg, Germany). The internal standard (IS) for the lipidomics analysis was EquiSPLASH Lipidomix (Avanti Polar, Alabaster, AL, USA): a mixture of 14 deuterated lipid molecules all at a concentration of 100 µg/mL. Deuterated Psychosine Quantitative Mass Spec Standard (Galactosyl(β) Sphingosine-d5; PSY-d5) with the concentration of 1 mM was purchased from Avanti Polar Lipids.

### 2.4. Sample Preparation for Lipidomics

Lipids were extracted from pellet according to the procedure of Pellegrino et al., with minor modifications [5]: 36 mL of the MMC extraction mixture (Methanol, MTBE, and CHCl_3_) was prepared by mixing 11.5 mL of methanol, 12 mL of MTBE and 12 mL of CHCl_3_, then adding 0.5 mL of (IS) diluted 1:10 in methanol and 125 ng/mL PSY-d5. Each sample was processed by adding 1 mL of MMC extraction mixture, vortexing for 5 s, immerging for 10 min in an ultrasonic bath, vortexing for 5 s again, then placing it in a Thermoshaker (Euroclone, Milan, Italy) and shaking for 20 min at 1500 rpm at 20 °C. The samples were subsequently subjected to centrifugation at 16,000× rcf for 10 min at 4 °C. The resulting supernatant was then transferred into a vial for the injection. A pool of all samples was extracted using the same procedure. An Eppendorf tube with only the extraction mixture MMC was prepared to use as a blank.

### 2.5. LC/MS Analysis

LC/MS analysis was performed using an Agilent analyzer, consisting of an Agilent 1260 Infinity II liquid chromatograph coupled with Agilent 6530 Accurate-Mass Q-TOF (quadrupole-time of flight) analyzer and an Agilent JetStream source. Liquid chromatographic separation was performed with an Ascentis express C18 (150 mm × 2.1 mm, 2.7 µm) Supelco column maintained at 50 °C with a flow rate of 0.25 mL/min.

We used the LC conditions as previously reported in the work of Alabed et al. [12] with minor modifications. Briefly, a quaternary gradient composed of 4 different solvents was used as a mobile phase for the chromatographic separation: water (A), acetonitrile (B), methanol (C), and isopropanol (D). The A, C, and D solvents were modified by adding ammonium fluoride (0.2 mM) and ammonium acetate (10 mM). The B solvent, on the other hand, was modified by only adding ammonium fluoride (0.2 mM). Check Table S1 for the gradient.

Raw data acquired under these conditions were used for both untargeted lipidomic profiling and targeted determination of PSY.

Gathered within the range of 50–3200 *m*/*z*, spectrometric data were acquired in both positive and negative polarities using the full-scan mode. The pooled sample was analyzed at least three times in an iterative data-dependent acquisition mode to collect the maximum number of MS^2^ spectra possible. The Agilent JetStream source was operated according to the following parameters: 280 °C for the nitrogen gas (N2), 12 L/min for the drying gas, 50 psi for the nebulizer, and 300 °C at 12 L/min for the sheath gas.

### 2.6. Raw Data Processing

The open-source program MS-DIAL (4.9) was used to conduct peak picking, alignment, and annotation on the raw data [25].

Following the guidelines of the Lipid Standard Initiative, the MS and MS^2^ were used to annotate the lipids at the molecular species level [26].

Lipid semi-quantification was conducted utilizing the deuterium-labeled internal standard (EquiSPLASH Lipidomix) according to Tsugawa H. et al. [25], for each lipid class at levels 2 and 3 of Lipidomics Standards Initiative recommendations; check Table S2. The semi-quantification on the raw data was carried out using an R script written in our laboratory based on the concentrations on the standards added to samples. The concentration of natural PSY in the samples was determined using the internal standard PSY-d5 added to each sample at a known concentration. At the end of the analytical workflow, a matrix for each tissue (B, C, SC, and SN) was obtained that contains the concentration in µg/µg of proteins of 246, 219, 236, and 220 and annotated lipid molecular species belonging to 19 lipid classes, respectively; see Supplementary Materials Tables S3–S6).

LipidOne [8] and a series of in-house R scripts were used to perform a multivariate statistical analysis on the dataset, for an in-depth analysis of lipid building blocks, for a chemoinformatic analysis, and to produce the graphs. Comparisons between groups following *t*-test or ANOVA are considered significant with a *p*-value < 0.05. PCA and PLS-DA analyses were performed on the data matrix after autoscaling or Pareto scaling.

## 3. Results and Discussion

The Lipidomic dataset of each tissue obtained from the lipidomic analysis contains qualitative and semi-quantitative information on all detected and annotated lipid molecular species.

To evaluate the variations in the lipid profiles in the four tissues, multivariate statistical data analysis was performed at the levels of lipid class, lipid molecular species, and lipid building blocks.

### 3.1. Within-Class Comparison of Lipid Profiles of Diseased and Healthy Mouse Models

The molecular species were grouped by class. In all four tissues, lipid molecular species belonging to 18 lipid classes were detected, except for the SC tissue in which 19 lipid classes were detected.

Table 1 shows the average concentrations of each class in the four tissues comparing WT vs. HOM. Concentrations are expressed in µg/µg of proteins. Tables for each tissue, complete with concentration, experimental error, *p*-value, and percentage composition, are given in Supplementary Tables S7–S10.

Looking at the total lipid concentrations shown in Table 1, it can be seen that there is a general decrease in the total lipid quantity within the HOM group; however, only the decrease in SN tissue is statistically significant with a *p*-value of 0.00296. In fact, the SN tissue in WT is very rich in lipids compared to the other tissues in WT. This can be explained by the fact that the myelin in peripheral nerve tissue contains more lipids [27]. In fact, the lipidomic analysis was carried out on the entire tissue, so not only do the cell membranes of all neural cells contribute to the lipid profile, but it is also worth considering the contribution of myelin and lipid rafts, enriched with some lipids such as galactosyl ceramide, sphingomyelin, and cholesterol [28,29]. The myelin sheath is an extensively elongated and specialized plasma membrane that envelops the nerve axon in a spiral manner. These myelin membranes are derived from and constitute the Schwann cells in the peripheral nervous system (PNS) and the oligodendroglial cells in the central nervous system (CNS).

From Table 1, it can be noticed that in the B tissue only the lipid class SHex-Cer is statistically different; in the C tissue three classes are different: PE-O, Hex-Cer, and SHex-Cer; in the SC tissue six classes are statistically different between WT and HOM: CE, DG, PE-O, LPI, PA, and PC; and lastly, in the SN tissue thirteen classes differ between HOM and WT: CE, DG, PC-O, PE-O, Hex-Cer, LPE, LPI, PA, PC, PE, PG, PI, and SHex-Cer.

The large difference between WT and HOM found in SN could be attributed to Krabbe disease, which is characterized by a profound neurodegeneration and demyelination of nerve tissue [30]. In fact, the classes most represented in the myelin sheaths (HexCer, SHexCer, PC, PE-O) are those most decreased in the SN of HOM [28]. 

Myelin is a fatty substance that plays a crucial role in insulating and protecting nerve fibers, allowing for efficient transmission of nerve impulses. Although myelin serves a comparable function in both the CNS and PNS, notable distinctions exist between them.

First, myelin in the CNS is primarily found on the axons of neurons within the brain and spinal cord, and the primary cells responsible for producing myelin in the CNS are oligodendrocytes. In the PNS, instead, myelin is present on the axons of neurons that extend outside the brain and spinal cord and is produced by Schwann cells. Additionally, while oligodendrocytes are responsible for myelinating multiple axons, Schwann cells alternatively myelinate a single axon each. 

Furthermore, and very importantly for our analysis, myelin in the CNS tends to have thinner layers and more internodes compared to in the PNS, whereas myelin in the PNS typically has thicker layers and fewer internodes, which can result in faster nerve signal propagation along peripheral nerves. This characteristic supports the hypothesis that we would fine more differences in the sciatic nerves compared to the CNS since myelin lipids may be the more abundant contributors to our sciatic nerve samples [31].

On the other hand, the reduction in specific lipid classes, such as PC, may be attributed to oxidation processes, leading to the production of oxidized PC (OxPCs). OxPCs are considered potent neurotoxic molecules and mediators of neurodegeneration within nerve tissues [32].

Surprisingly, no significant differences were found between the amounts of sphingomyelins between HOM and WT in any of the four tissues, despite the fact that the SM class is characteristic of the myelin sheath [28]. As already stated, there are no previous studies on the variation in the lipid profile between WT and HOM to be used as comparisons.

**Figure 1:**
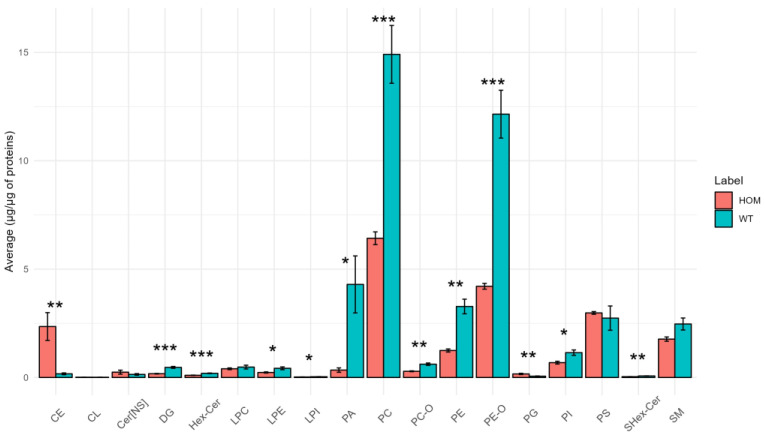
Bar graph showing the average concentration of each lipid class in SN tissue in the HOM vs. WT groups. The bars represent the experimental error (*n* = 5 WT, *n* = 4 HOM). Asterisks represent *p*-values following *t*-test (* *p* < 0.05, ** *p* < 0.01, *** *p* < 0.001).

SN tissue is the one in which the most differences were found at the level of lipid classes (13 out of 18 classes have significant differences between the two groups), which we present in a bar chart in Figure 1.

As depicted in Figure 1 above, significant differences are evident among the classes (DG, PC-O, PE-O, Hex-Cer, LPE, LPI, PA, PC, PE, PI, and SHex-Cer), where HOM demonstrates lower abundances of these classes compared to WT. However, in the case of the classes CE and PG, HOM exhibits higher abundances compared to WT. 

An unexpected result is the low levels of HexCer and SHexCer; this is not in agreement with the data reported in the literature, according to which the two lipid classes should account for approximately 20% and 4% of the total lipids in the myelin sheath [33]. A possible technical explanation for this result is that in this lipidomics study HexCer and SHexCer were normalized using the deuterated standard ceramide, which might have a different response in the instrument.

Furthermore, referring to Figure 1, it can be seen that the concentration of DGs in the SN tissue also changed considerably. DGs are an important lipid class, and are an essential intermediate for the synthesis of the triglycerides (TG) and some phospholipids. In addition, they work as second messengers that represent a small portion of the membrane but can alter the properties of the membrane itself, for example by promoting membrane fusion and fission [34,35].

Moreover, as already mentioned, there are important differences in the class of PE-Os, which are a fundamental class, together with the other ether phospholipids, for the proper development and functioning of the nervous system. In fact, they are involved in many cellular functions, e.g., signaling mechanisms, antioxidative defense, and membrane structure and function, but particularly in nervous tissue they are crucial for synaptic transmission, myelin formation and its maintenance, and play a role in neuroinflammation [36]. Notably, increased levels of CE have also been observed in other neurodegenerative diseases [37].

### 3.2. Interactions between Lipid Classes

Enzyme activity influenced by the study conditions can be predicted by studying the weight vectors (ratios of products to reactants) of well-known transformations among the lipid classes [38]. *p*-values generated via the *t*-test are used to select significant active or suppressed reactions.

**Figure 2:**
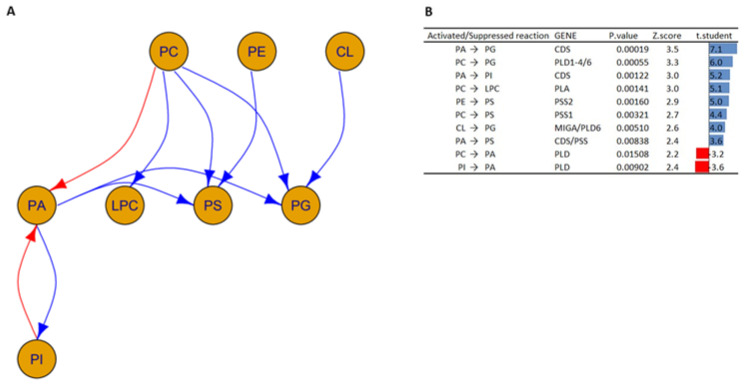
(**A**) A network graph displays the possible transformation of lipid classes in the HOM group compared to the WT group, which can be explained using established biochemical processes as a reference. The arrows show the activated (blue) and suppressed (red) transformations. (**B**) Table shows the activated and suppressed transformations between lipid classes, each with the possible enzyme and coding gene involved and the associated *p*-value, Z-score, and t-student value. Blue and red bars represent activated and suppressed reactions, respectively. The *t*-test is based on the values of the ratios of products to reactants between HOM and WT.

The graph shown in Figure 2 was created by analyzing the SN data matrix following the same procedure as shown in Husam B. R. Alabed et al.’s work [12].

The study of transformation among the lipid classes illustrated in Figure 2 shows a complex network of interactions.

In particular, the following interactions between the HOM group versus the WT group with *p*-values < 0.02 are highlighted and suggest the following:

1. Increased activity of the enzyme phosphatidate cytidylyltransferase (also known as CDP-diacylglycerol synthase), which is involved in the transformation of PA to PG and PA to PI [39].
2. Decreased enzyme activity of phospholipases D (encoded by *PLD*), which is responsible for the transformation of PI into PA and PC into PA [40].
3. Increased activation in the phosphatidylserine synthase 1 and 2 enzymes (encoded by *PSS1* and *PSS2*, respectively), which are responsible for the transformation of PC and PE into PS [41]. In addition, the concomitant activation of the PSS together with the CDS results in the transformation of PA into PS.
4. Increased activation of the enzymes phospholipase D1 (encoded by *PLD1*), 5′-3′ exonuclease PLD4 (encoded by *PLD4*), and mitochondrial cardiolipin hydrolase (encoded by *PLD6*), which underlies PG synthesis from PCs [40].
5. Increased activation of the enzymes mitoguardin 1 (encoded by *MIGA*) and mitochondrial cardiolipin hydrolase (encoded by *PLD6*), which are involved in the transformation of CL into PG.
6. Increased activity of the phospholipase A enzyme (encoded by *PLA*), which is responsible for the conversion of PCs into LPCs [40]. This is in agreement with what has been reported in the literature; in fact, a similar trend has also been found in oligodendrocytes and astrocytes, where psychosine influences the regulation of cell death through the formation of LPCs [42,43].

The data obtained in this study can allow us to hypothesize an increased activity of CDS and PSS enzymes in the Twitcher murine model studied, while the regulation of phospholipase such as PLD and PLA could be altered in both negative and positive ways in the Twitcher murine model.

The results obtained at the level of lipid classes suggest that the differences found in lipid profile could be attributed to both myelin degradation and drastic changes in cellular metabolism between the two groups. The latter may be the basis for changes in the structure of cell membranes.

### 3.3. Analysis at the Level of Lipid Molecular Species

At the level of lipid molecular species, a minimum of 219 to a maximum of 246 individual lipid molecules were revealed in the four tissues analyzed (Tables S1–S4).

To explore the data, unsupervised multivariate chemometric statistical methods, such as principal component analysis (PCA), can be used for an in-depth examination of the data matrices of the experimental samples analyzed.

**Figure 3:**
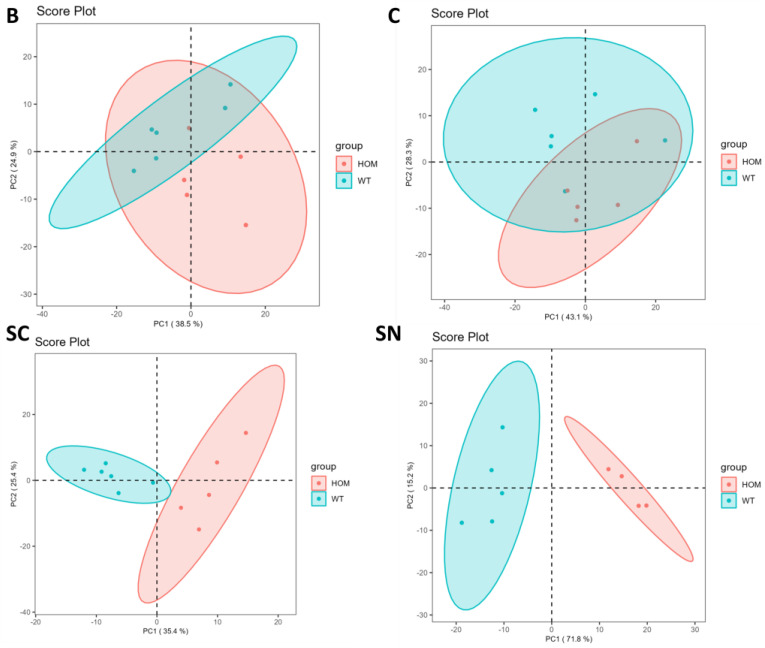
Principal component analysis of lipid molecular species in HOM and WT of B (brain), C (cerebellum), SC (spinal cord), and SN (sciatic nerve).

Figure 3 demonstrates the PCA performed by using LipidOne and R scripts written in our laboratory after normalization of the data matrix by median and scaling with a Pareto scaling algorithm. The ellipses enclose the scores inside a region with 95% confidence.

PCA from Figure 3 confirms what has already been seen in the lipid class analysis: two clusters of comparison between HOM and WT in both cerebellum tissue and brain tissue are clearly overlapping, while in spinal cord tissue two cluster are slightly overlapping. Only in sciatic nerve tissue is there a clear cluster, and the SN plot in Figure 3 shows two clearly detached clusters; the first component, which explains the 71.8% of the variance, distinguishes the WT group from the HOM group.

**Figure 4:**
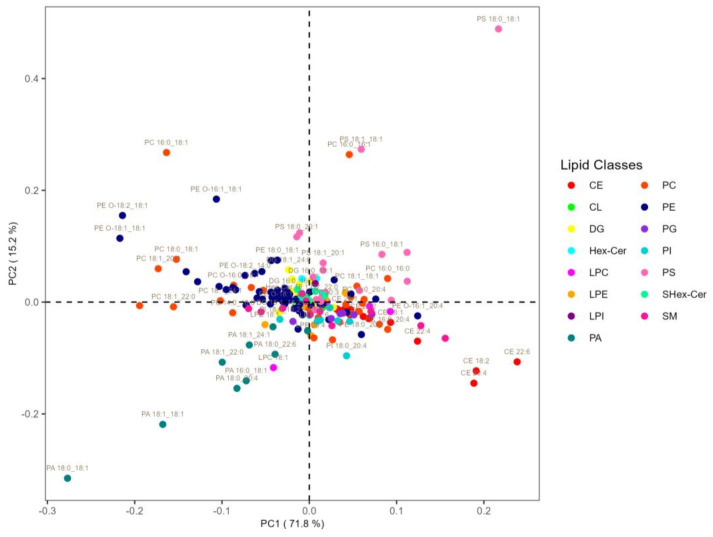
Loadings plot of the principal component analysis (PCA) results of SN tissue. The color shows the different lipid classes.

Since the analysis at the level of molecular species also demonstrated that the SN tissue has the most differences, we decided to focus our analysis on SN tissue. Figure 4 shows a loading plot of PCA conducted only on SN tissue to highlight the variables that give the largest contribution to the components.

Since the first component causes a separation of the clusters to the right and left of the score plot, the variables underlying the separation between the two clusters are to be found at the extreme right and left of the loading plot. One notices a prevalent clustering on the left of the molecular species belonging to the Ether-PE (dark blue) and PA (dark green) classes, and a prevalent clustering on the right of the PS (light pink) and CE (red) classes.

A closer look at the molecular species belonging to the PE class on the left side of the loading plot shows that they are of the ether type. Specifically, the variables that contribute most to the separation of the two groups are Ether PE 16:1_18:1, Ether PE 18:2_18:1, Ether PE 18:1_18:1, PA 18:0_18:1, PA 18:1_18:1, PA 18:1_22:0, PA 18:0_20:4, and PA16.0_18:1 on the left side, and CE 20:4, CE 18:2, CE 22:6, PS 18:1_18:1, and PS 18:0_18:1 on the right side.

**Figure 5:**
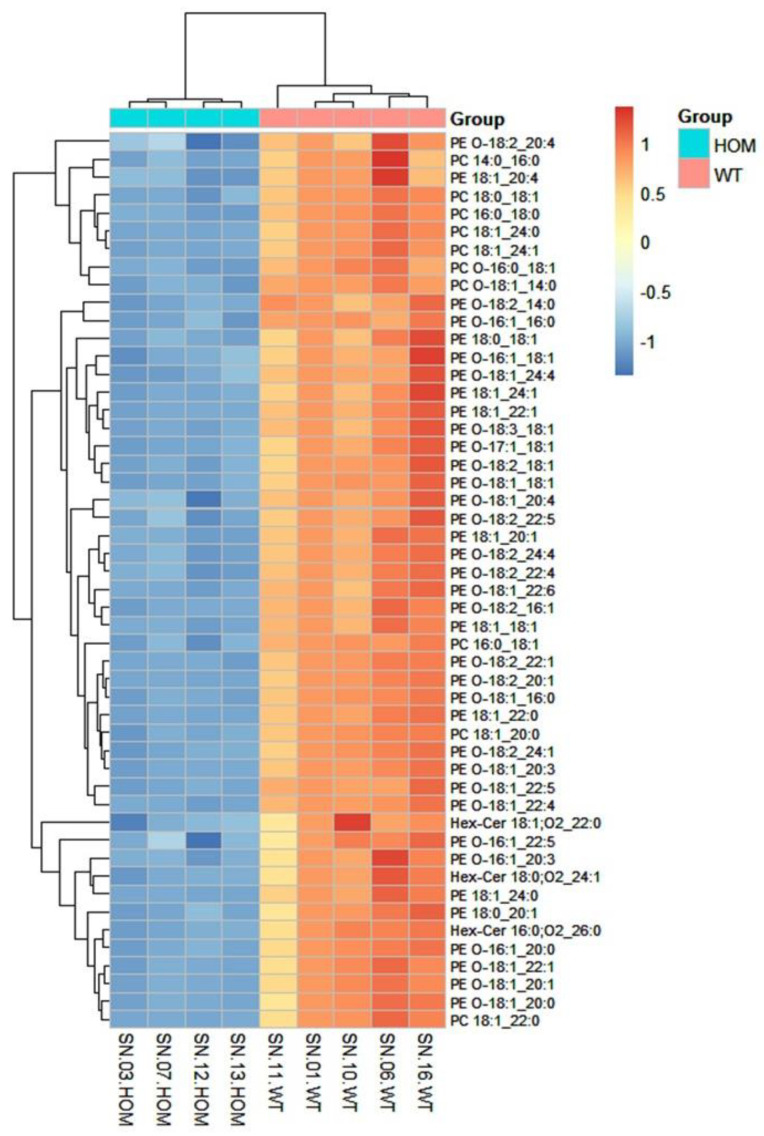
Heatmap: This figure illustrates the correlation among the 50 most significant lipid molecular species within each sample. Colors represent the expression level of each individual species in each sample based on their concentration. The analysis employed the Euclidean correlation index and the complete clustering model.

The heatmap analysis was performed by reporting the data of the 50 lipids found to be most significant following a *t*-test (Figure 5). 

The heatmap in Figure 5 shows that among the 50 most significant lipid molecular species, many molecular species of PE-O, PE, PC, and Hex-Cer have lower concentrations in the HOM group compared to the WT. The results obtained are in agreement both with those obtained by analyzing the lipid classes and with the data available in the literature. In fact, as already highlighted earlier, they are lipid classes present in myelin [44].

**Figure 6:**
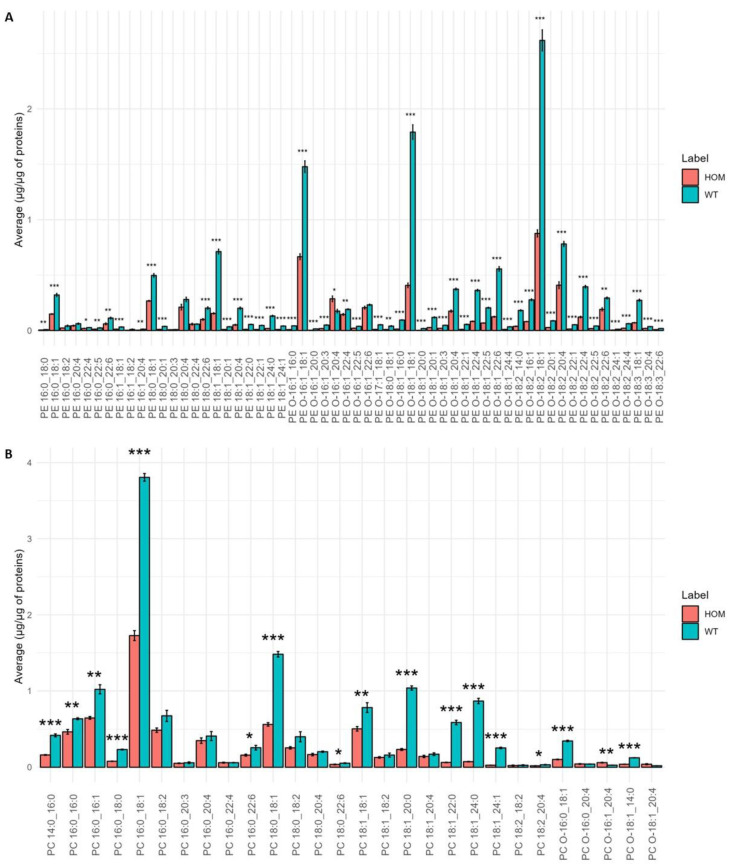
Bar graph of the average concentration of each molecular species belonging to the lipid classes PE (**A**) and PC (**B**) in SN tissue of the HOM vs. WT groups. The bars represent the experimental error (*n* = 5 WT, *n* = 4 HOM). Asterisks represent *p*-values following *t*-test (* *p* < 0.05, ** *p* < 0.01, *** *p* < 0.001).

Since the heatmap showed a clear level of significance between the PE and PC molecular species, a detailed analysis of PE and PC molecular species was carried out. Figure 6A,B show the result of this analysis.

The PE profile (Figure 6A) shows a significant decrease in all molecular species in the HOM group compared to WT. The PC profile (Figure 6B) shows that many molecular species are significantly decreased in the HOM group compared to WT, while some molecular species such as PC O-16:1_20:4 significantly increase in HOM. A very pronounced decrease can be seen for molecular species with saturated fatty acids of 20, 22, and 24 carbon atoms.

#### Psychosine Concentration

**Figure 7:**
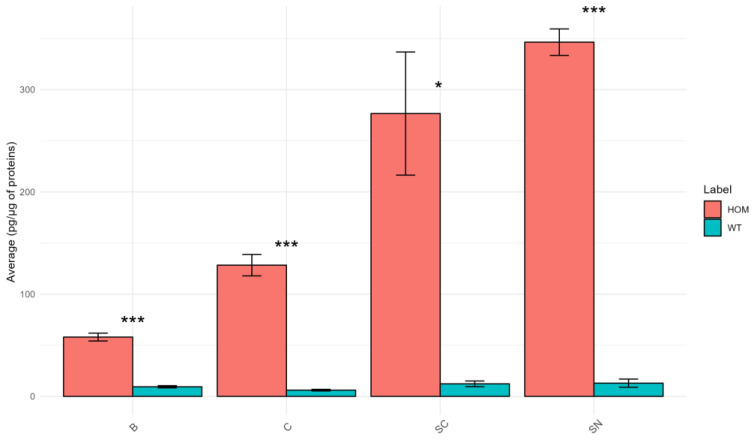
Bar graph shows the mean concentration of psychosine in the HOM vs. WT groups in B, C, SC, and SN tissues. The bars represent the experimental error (*n* = 6 WT, *n* = 5 HOM). Asterisks represent *p*-values following *t*-test (* *p* < 0.05, *** *p* < 0.001).

The results of PSY concentration are illustrated in Figure 7.

The graph in Figure 7 shows that the concentration of PSY differs in a statistically significant manner in all tissues considered. As expected, all HOM tissues show a very high concentration of PSY compared to WT. This is particularly evident in SC and SN tissues where the HOM/WT ratio for PSY is approximately 22 and 27, respectively. PSY accumulation has already been reported in the literature to be greater in sciatic nerve tissue than in brain tissue [1].

This result is related to the finding that SC and especially SN are the tissues that show a greater difference in lipid profile in the HOM group compared to the WT group. Regarding the SN, this finding is in agreement with the fact that an extensive PNS pathology is more characteristic of TWI compared with Krabbe disease patients [45]. Accordingly, an increased concentration of PSY has been already reported in the SN of TWI mice compared to CNS organs [46,47].

The PSY pathway is directly related to certain lipid classes identified in this study, such as Cer and Hex-Cer. Consequently, the defect in the GALC gene could potentially interfere with the concentrations of these lipids within HOM mice (see Figure S1). Moreover, PSY was found to modify the membrane by altering sphingomyelin-enriched domains, increasing the stiffness of certain localized areas and promoting shedding of membrane microvesicles; the same effects were also found in the myelin sheath [48]. 

Correlation analysis was also conducted between the concentration of PSY and the concentration of other lipid classes (see Table S11). It is notable that most of the lipid classes, such as most of the phospholipids, SM, Cer, Hex-Cer, SHex-Cer, and DG, are negatively correlated with PSY. Conversely, CE and PG show a positive correlation with PSY.

### 3.4. Analysis of Lipid Building Blocks

LipidOne was also used to perform an in-depth analysis of the building blocks of the lipids (fatty acid chains inside the molecules). By using an R script written in our laboratory, it was possible to highlight the significant differences between the two groups at the level of length and the amount of unsaturated fatty acids making up the lipids. It was also possible to perform a network analysis by studying the weight vectors of the transformations between the chains to illustrate the significant transformations among the various chains and predict the genes and enzymes involved in these transformations inside the cause group (HOM) based on well-known transformations and interactions reported in the literature.

**Figure 8:**
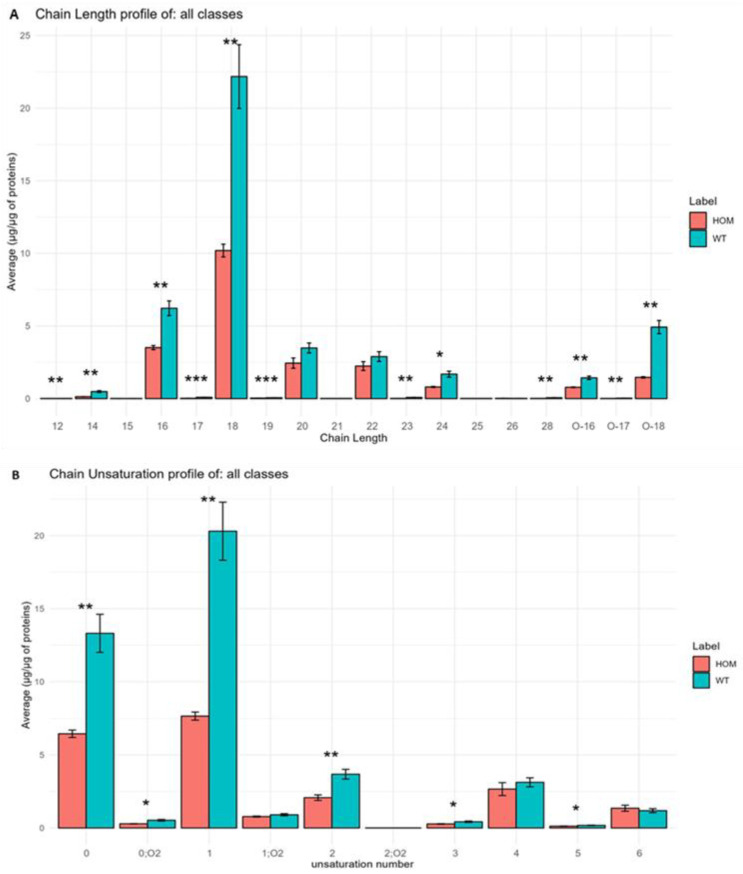
(**A**) Histograms obtained using LipidOne represent the results of the analysis at the level of fatty acid chain length in SN tissue in the HOM group compared to WT group. (**B**) Histograms obtained using LipidOne represent the results of the analysis at the level of chain unsaturation in HOM group compared to WT group. Asterisks represent *p*-values following *t*-test (* *p* < 0.05, ** *p* < 0.01, *** *p* < 0.001).

Regarding the study of chain length and unsaturation, as shown in Figure 8, our results are in agreement with what has been observed in other studies [49]: chains are mainly composed of 16 and 18 carbon atoms. Meanwhile, at the level of unsaturation, we found that the most present chains are those with one unsaturated or non-unsaturated chain.

In Figure 8A, it is possible to note that most of the chain lengths are statistically different; in fact, we can note a general decrease in all these chains inside HOM. The same results can be observed in Figure 8B, and demonstrate the amount of unsaturation inside the building blocks of the lipid inside the HOM group compared to the WT group.

**Figure 9:**
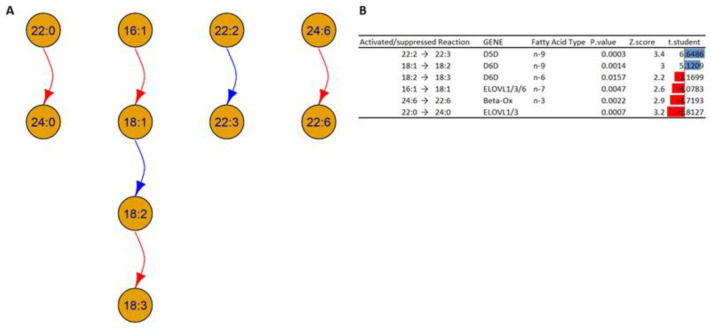
(**A**) A network graph shows the transformation of the buildings block in the HOM group compared to the WT at the chain level and unsaturation level of fatty acids inside all 18 lipid classes. The arrows show the activated and suppressed transformations. (**B**) Table shows the activated and suppressed transformations between lipid building blocks, each with the possible enzyme and coding gene involved, and the associated *p*-value, Z-score, and t-student value. Blue and red bars represent activated and suppressed reactions, respectively. The *t*-test is based on the values of the ratios of products to reactants between HOM and WT.

The network analysis was performed at the level of building blocks to study the possible transformation of the lipid chains and the possible enzymes involved in these pathways. The network graph in Figure 9 illustrates the result.

In Figure 9, the analysis of building blocks in HOMs compared to WTs shows two types of interconversions:

1. Desaturation of the fatty acids: Based on the analysis, it is possible to note an increase in the concentrations of the chain 18:2 in HOMs compared to WT; this could be due to an alteration in the activity of Δ6 desaturase (D6D), which could indicate an increase in the transformation of fatty acid 18:1 (18 carbon atoms with one unsaturation) to fatty acid 18:2, and/or a decrease in the transformation of fatty acid 18:2 to 18:3. In addition, the analysis shows an increase in fatty acid 22:3 in HOM mice, which could indicate an increased desaturation of fatty acid 22:2, which could indicate an alteration in the activity of Δ5 desaturase (D5D).
2. Elongations and beta oxidation of the fatty acids: The analysis suggests a decreased activation in the activity of specific elongases (ELOVL) such as ELOVL1, ELOVL3, and ELOVL6, which can explain the decrease in the transformation of fatty acids 16:1 to 18:1 and 22:0 to 24:0 inside HOMs compared to WTs. The analysis also suggests decreased activation of the beta oxidation of fatty acid 24:6 in the HOMs.

The observed data generally suggests an altered regulation of the *D6D* gene or the respective enzyme; this can be due to activation or suppression depending on the subcellular compartment. At the same time, the data show a suppression of the enzymes encoded by the *ELOVL* genes and an alteration in beta oxidation.

**Figure 10:**
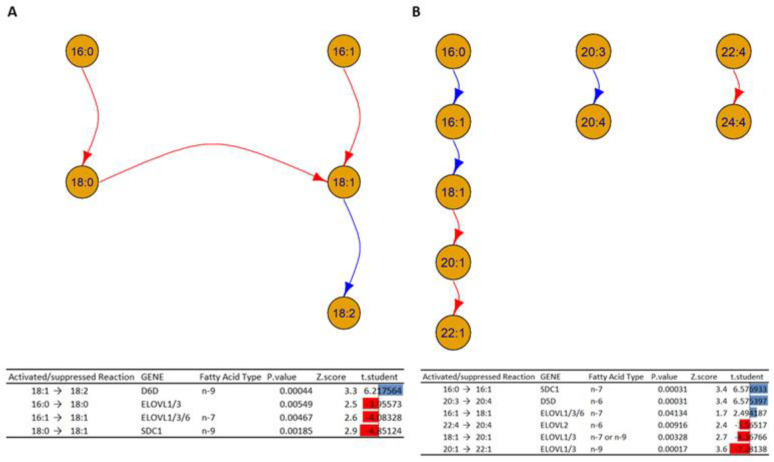
Network graphs depicts the interconversion of the building blocks in the PC (**A**) and PE-O (**B**) lipid classes in the HOM group compared to the WT. Table shows the activated and suppressed transformations between lipid building blocks, each with the possible enzyme and coding gene involved, and the associated *p*-value, Z-score, and t-student value. Blue and red arrows and bars represent activated and suppressed reactions, respectively.

Since PCs and PE-Os are the most altered lipid classes, a building block study was also carried out for each of these phospholipids. The results are summarized in Figure 10.

Figure 10 shows the interconversions between building blocks in the lipid classes PC (A) and PE-O (B). In particular:

1. The analysis in Figure 10A shows a decrease in activity in the elongation of the fatty acids 16:0 to 18:0 and 16:1 to 18:1, which could indicate alterations in the enzymes ELOVL1, ELOVL3, and ELOVL6 inside HOMs. On the other hand, the desaturation of 18:1 to 18:2 is increased in HOMs, which could indicate an increased activity of D6D. In addition, the analysis suggests an alteration in the activity of the enzyme Stearoyl-CoA desaturase (*SCD1*), which can explain the decrease transformation of the fatty acid 18:0 to 18:1 in the HOMs.
2. Figure 10B shows a cascade of reactions that favor the formation of fatty acid 18:1 from the unsaturation of fatty acid 16:0, which could indicate an increased activity of SDC1 and a subsequent elongation to fatty acid 18:0, mediated by the ELOVL 1/3/6 genes. At the same time, the elongation of fatty acids 18:1 to 20:1 and 20:1 to 22:1 is inhibited, which could suggest alterations in the enzymes ELOVL 1/3. In addition, the desaturation of fatty acids 20:3 to 20:4 could suggest increased activation.

To the best of our knowledge, there are no authors reporting similar results in the literature or gene expression and enzyme activity studies confirming the results based on the analysis of the lipid phenotype obtained in this work.

## 4. Limitations

It must be considered that this study has limitations. Firstly, its exploratory nature results in a limited number of samples, due to the difficulty in obtaining tissues from the animal model. In fact, obtaining tissues from Twitcher mice is challenging due to maintenance issues. Often the homozygous mutant dies shortly after birth or is sacrificed directly by the mother. Thus, obtaining homozygous mutants that can survive for use in experiments is not easy. Additionally, there are approximately 10% dropouts due to disease complications. Moreover, they are considered a suffering model under Italian law, limiting the number of animals for experiments. Furthermore, the scarcity of samples did not allow us to consider additional parameters that could influence the lipid profile of nervous system tissues, such as the sex, dietary behavior, and body mass index of both healthy and diseased mice.

A further limitation is that the untargeted lipidomic approach detected the presence of lipid classes and subclasses, which are not present as marked species in the internal standard used for annotation (Splash Lipidomix). This may have resulted in an over- or under-estimation of these classes (such as PE-O, Hex-Cer, SHex-Cer).

## 5. Conclusions

In this work, we analyzed four different tissues belonging to the nervous system (brain, cerebellum, spinal cord and sciatic nerve) of healthy (WT) and Krabbe disease (HOM) mouse models to highlight any changes in the lipid profile attributable to the disease.

The results of the lipid profile analysis revealed 219 to 246 molecular species belonging to up to 19 lipid classes in the different tissues.

The data show that the total lipid concentration decreases in all tissues in the HOM group compared to the corresponding tissues in the WT group; however, this decrease is statistically significant only in the sciatic nerve tissue, where the total lipid concentration is almost halved in the HOM group compared to the WT.

Regarding the lipid class analysis, the brain demonstrated less differences between the two groups, with just 1 class differing between the two groups; on the other hand, SN demonstrated differences between the two groups in 13 different lipid classes. 

At the level of lipid molecular species, the PCA carried out on all four tissues demonstrated the same result with the most separation between the two groups observed in the SN and SC tissues. In addition, the concentration of psychosine in the four tissues increases in HOM compared to WT and correlates with the magnitude of the change in lipid profile in the four HOM tissues.

The differences in the lipid profile are particularly pronounced in SN tissue. The lack of a reference for the lipid profile of the sciatic nerve does not allow a comparison of the data we obtained. However, using the typical myelin lipid profile as a reference, it can be seen that the lipid classes most decreased in HOM are those characteristic of the myelin sheath (such as HexCer, SHexCer, PC, and PE-O). In contrast, no major differences in SM levels were found. The concentrations of HexCer and SHexCer were lower than expected in both HOM and WT. It is also important to mention that the decrease in the concentration of PC within the HOM could be the result of oxidation processes affecting these species. This process produces a specific oxidized PC, which, in turn, could contribute to increased neurodegeneration within nerve tissues. In addition, CE levels were increased in the HOM group compared to the WT, which could be a compensating system to reduce cholesterol accumulation caused by the degradation of the myelin. These results not only demonstrate that these changes could be the consequence of neurodegeneration but also could be its cause.

Focusing on the SN tissue, the heatmap shows a number of molecular species which differ between the two groups, most of them of the PE-O and PC classes, such as PE O-16:1_18:1, PE O-18:1_18:1, PE O-18:2_18:1, PE O-16:1_18:1, PC 16:0_18:1, and PC 18:0_18:1.

The analyses of lipid class interactions showed that the transformation of Pas and PCs into PG, PI, and PS is favored. This allows the assumption that there is an altered regulation of genes such as CDS, PLD, and PSS. At the level of the buildings blocks analysis, a general inhibition of the elongation of carbon chains can be seen, while the activity of desaturase enzymes increased, mainly involving chains with 18 and 22 carbon atoms. These results could indicate alterations in specific enzymes involved with the elongation and desaturation of fatty acids such as elongase 1, elongase 3, elongase 6, Δ6 desaturase, Δ5 desaturase, and Stearoyl-CoA desaturase.

These changes confirm the presence of significant changes and differences in the lipid profile of specific tissues inside the HOM mice compared to WT. These changes can be helpful to increase knowledge of the molecular causes of Krabbe disease. In fact, in a systems biology scenario, the changes in the lipidic phenotype highlighted in this study could allow us to hypothesize and predict the alterations in the enzymatic activity and genetic expression that could be caused by the disease. Regarding the possible clinical significance of the differences in the lipid profile in KD patients, it may help, first of all, in the identification of early biomarkers for KD, which are currently missing at the moment in clinical practice. Additionally, some lipid biomarkers could help to identify the degree of progression of the disease, and thus the more appropriate approach to treat specific patients. Also very appealing is the possible usage of lipid biomarkers as a readout of the effect of a therapeutic approach. Classic biomarkers of the disease, such as GALC enzymatic activity and PSY levels, are not enough to study KD. Indeed, Twitcher mice treated with experimental therapies (as gene therapy approaches) that present GALC enzymatic activity and PSY levels in normal ranges are not cured from KD [47,50], but die from multiple and still-unclear complications.

## Figures and Tables

**Table 1:** Summary table showing the total concentration and the mean concentration of each lipid class for each tissue in the WT and HOM groups compared. For each class, *p*-values are given following *t*-test (* *p* < 0.05, ** *p* < 0.01, *** *p* < 0.001).

	B			C			SC			SN		
Explained Class Name	WT	HOM	*p*-Value	WT	HOM	*p*-Value	WT	HOM	*p*-Value	WT	HOM	*p*-Value
Cholesteryl ester	0.087	0.115		0.131	0.182		0.137	0.730	*	0.169	2.351	**
Ceramide	0.022	0.023		0.055	0.050		0.063	0.054		0.011	0.012	
Cardiolipin	0.330	0.327		0.182	0.203		0.143	0.217		0.142	0.246	
Diacylglycerol	0.115	0.101		0.210	0.137		0.445	0.251	*	0.466	0.174	***
Ether-linked phosphatidylcholine	0.077	0.076		0.908	0.863		0.245	0.218		0.613	0.287	**
Ether-linked phosphatidylethanolamine	3.670	3.483		5.619	4.557	*	10.399	8.169	*	12.154	4.209	***
Ether-linked phosphatidylinositol							0.198	0.176				
Hexosylceramide	0.029	0.023		0.040	0.028	*	0.151	0.128		0.193	0.102	***
Lysophosphatidylcholine	0.231	0.239		0.251	0.262		0.598	0.505		0.480	0.401	
Lysophosphatidylethanolamine	0.406	0.409		0.380	0.405		1.065	0.853		0.424	0.232	*
Lysophosphatidylinositol	0.029	0.024		0.042	0.033		0.054	0.040	*	0.035	0.023	*
Phosphatidic acid	0.052	0.046		0.158	0.092		2.891	1.065	*	4.295	0.342	*
Phosphatidylcholine	8.254	7.902		8.312	7.994		9.829	8.323	*	14.912	6.426	***
Phosphatidylethanolamine	2.612	2.590		3.254	2.822		4.621	3.724		3.277	1.245	**
Phosphatidylglycerol	0.082	0.089		0.096	0.097		0.184	0.227		0.059	0.164	**
Phosphatidylinositol	0.438	0.364		0.925	0.817		1.421	1.194		1.146	0.684	*
Phosphatidylserine	3.071	3.009		4.182	3.832		6.569	5.589		2.740	2.980	
Sulfatide	0.021	0.018	*	0.032	0.023	***	0.058	0.044		0.070	0.037	**
Sphingomyelin	0.614	0.617		0.186	0.194		0.893	1.056		2.468	1.771	
Total	20.139	19.456		24.962	22.591		39.964	32.564		43.654	21.686	**

## Data Availability

See Supporting Information.

## Supplementary Materials

The following supporting information can be downloaded at https://www.mdpi.com/article/10.3390/biom13101562/s1: Figure S1: Metabolic pathway of GALC gene substrates; Table S1: Gradient table for liquid chromatographic separation; Table S2: Table for the standard used to semi-quantify each of lipid classes; Table S3: Information table of all lipid classes in Brain tissue; Table S4: Information table of all lipid classes in Cerebellum tissue; Table S5: Information table of all lipid classes in Spinal Cord tissue; Table S6: Information table of all lipid classes in Sciatic Nerve tissue; Table S7: Concentration table (in µg/µg of protein) of the lipid molecular species annotated in Brain tissue; Table S8: Concentration table (in µg/µg of protein) of the lipid molecular species annotated in Cerebellum tissue; Table S9: Concentration table (in µg/µg of protein) of the lipid molecular species annotated in Spinal Cord tissue; Table S10: Concentration table (in µg/µg of protein) of the lipid molecular species annotated in Sciatic Nerve tissue; Table S11: Correlation Table between PSY concentration and each lipid class annotated and semi-quantified.
