# Supplementary material for: Untargeted Lipidomic Approach for Studying Different Nervous System Tissues of the Murine Model of Krabbe Disease
Source: Biomolecules. 2023 Oct 23;13(10):1562. doi: 10.3390/biom13101562 (PMC10605133; doi:10.3390/biom13101562)
Supplement: Supplementary file 1 [file biomolecules-13-01562-s001.zip › Supplementary Materials Figure S1.pdf]

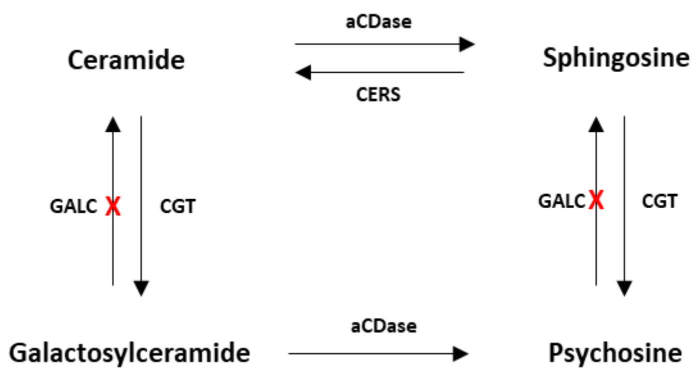

Figure S1: Metabolic pathway of GALC gene substrates. Sphingosine is first acylated to Ceramide, which is converted to Galactosylceramide by Ceramide Galactosyltransferase (CGT). The Galactosylceramide is considered a type of Hex-Cer. The CGT enzyme can also galactosylate the sphingosine directly to form Psychosine. Both Galactosylceramidase and Psychosine are degraded by GALC, which is absent in HOM mice of this study.
